# Development of an Estimation Instrument of Acoustic Lens Properties for Medical Ultrasound Transducers

**DOI:** 10.1155/2017/6580217

**Published:** 2017-12-22

**Authors:** Hojong Choi, Jongseon Johnson Jeong, Jungsuk Kim

**Affiliations:** ^1^Ultrasound System Hardware Laboratory and Department of Medical IT Convergence Engineering, Kumoh National Institute of Technology, 350-27 Gumi-Daero, Gumi, Gyeongbuk 35029, Republic of Korea; ^2^IMP System, 301-3ho 267-96 Gongdan2-dong, Gumi, Gyeongbuk 39373, Republic of Korea; ^3^Department of Biomedical Engineering, Gachon University, Incheon 21936, Republic of Korea

## Abstract

In medical ultrasound transducers, the transmission mode (pass-through) approach has been used to estimate the characteristics of the acoustic lens. However, it is difficult to measure the acoustic lens properties with high precision because of human, systemic, or mechanical measurement errors. In this paper, we propose a low-cost estimation instrument for acoustic lens properties connected with a customized database. In the instrument, three-axis and one-axis transmitting and material fixtures accurately align the transmitting and receiving transducers separately. Through the developed instrument, we obtained a precise standard deviation of the attenuation coefficient and velocity of the acoustic lens material of 0.05 dB/cm and 2.62 m/s, respectively. Additionally, the simultaneous alignment between the fixtures is controllable with developed programs, thus generating very accurate information of the acoustic lens about the testing ultrasound transducer. In our instrument, the database could support users in managing the result data efficiently. User programs developed using LabVIEW provide the capability to obtain precise values of the attenuation coefficient and velocity, which represent the fundamental material characteristics of the acoustic lens of the medical ultrasound transducers. The developed review program of the customized database can also search the acoustic lens information and store the experimental results.

## 1. Introduction

Medical ultrasound instruments have been widely used for nondestructive testing, medical imaging modalities, and sound navigation and ranging applications [1–4]. Medical ultrasound instruments are typically composed of medical imaging systems and ultrasound transducers [5–7]. Single- or multiple-pulse electrical signals generated from high-voltage pulse generator electronics are transmitted into the ultrasound transducers that generate the acoustic powers into the target medium [8]. The reflected and scattered acoustic signals should be received by the medical ultrasound transducers in order to be processed in the medical imaging systems [9]. The medical imaging systems consist of a beam former, signal processor, and image display [5]. In the medical imaging systems, the weak echo signals detected by the medical ultrasound transducers are processed through the signal processor and image processor. Finally, the images are shown on the image display, in order to ascertain the structural information of the target [10, 11].

In medical ultrasound instruments, the medical ultrasound transducers are one of the most important devices that convert the electrical pulse signals into acoustic signals or vice versa [12]. The medical ultrasound transducers affect the performance of the medical imaging systems [10, 13].

**Figure 1:**
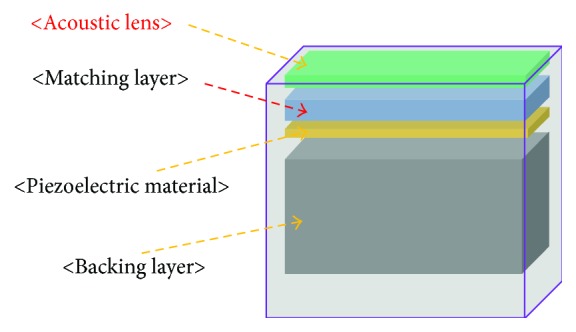
Structure of the acoustic lens in the medical ultrasound transducers.

Figure 1 illustrates the fundamental structure of the medical ultrasound transducers [14]. The acoustic lens is used to focus the ultrasound beam at the target distance and usually protects the matching layer because a medical ultrasound transducer without an acoustic lens could directly contact the target [15, 16]. The matching layer, which is one of the most important acoustic materials in the medical ultrasound transducer, is used to propagate as much ultrasound energy as possible, by matching the target medium with a piezoelectric material [17]. The piezoelectric material is used to convert electrical energy into ultrasound energy or vice versa [5]. The backing layer is used to absorb the ultrasound energy toward the medical imaging systems [2, 5].

As described previously, mechanical materials such as the acoustic lens, matching layer, and backing layer affect the performances of the medical ultrasound transducers. The acoustic impedance of the material is the mechanical impedances of certain materials inside the medical ultrasound transducers, and this value represents the amount of acoustic (ultrasound) energy transmitted or reflected through a certain medium towards the target [2].

The velocity of the piezoelectric materials is used to find the acoustic impedance of the materials, which is obtained by multiplying the density by the velocity, as described by Dowsett et al. [18].

(1) $$\begin{array}{c} AI=D*V, \end{array}$$

where AI is the acoustic impedance of the material and *D* and *V* are the density and velocity of the material, respectively.

The ultrasound signal is assumed to be significantly attenuated if the medical ultrasound transducers have a large acoustic impedance, because of the acoustic impedance mismatch with the transmitting medium such as water, air, or oil [10]. The acoustic impedance mismatch between the acoustic lens and matching layer also significantly deteriorates the ultrasound velocity, increasing the energy loss of the medical ultrasound transducers [19]. The attenuation coefficient of the acoustic materials can help estimate the amount of transmitting ultrasound waves absorbed in certain media [19]. In order to estimate the performance with several piezoelectric materials before the transducer fabrication, the medical ultrasound transducer designer uses simulation programs such as PiezoCAD, PZFlex, and Field II [20, 21]. The velocity and attenuation coefficient are among the important mechanical parameter factors to estimate the medical ultrasound transducer performance in simulation programs [2, 19]. In order to obtain accurate data of the velocity and attenuation coefficient, we constructed a database-supported estimation instrument of acoustic lens properties.

In this instrument, we built fixtures to hold the transducers and testing acoustic lens materials to obtain the material parameters with the transmission method. Each fixture provides the capability to compensate the nonalignment between the transmitting and receiving transducers. Temperature control is also utilized in order to maintain a constant ultrasound velocity. The instrument also enables users to obtain and store the velocity and attenuation coefficient data automatically in order to reduce human error and help users efficiently search for data in the instrument database. In addition, simultaneous alignment between the fixtures is controllable with developed programs. Therefore, our developed instrument can provide very accurate alignment of the fixtures, thus generating accurate information of the acoustic lens.

## 2. Method

**Figure 2:**
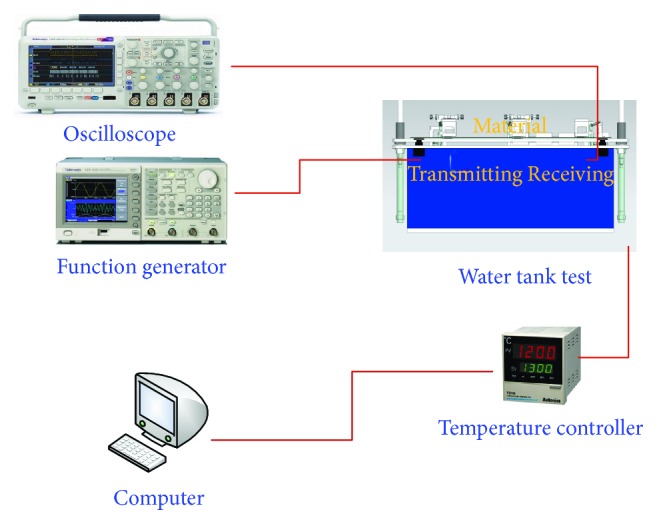
Block diagram of the developed estimation instrument of acoustic lens properties for medical ultrasound transducers.

Figure 2 illustrates the block diagram of the developed automatic database-supported estimation instrument of the acoustic lens properties for medical ultrasound transducers. First, the acoustic (ultrasound) waves generated by the transmitting transducers cannot be accurately detected by the receiving transducers because of the mechanical error of the fixtures or internal torsion of the constructed instrument. Therefore, transmitting, receiving, and material fixtures are provided to align both transmitting and receiving transducers together. Second, the ultrasound wave velocity is related to the water temperature; therefore, a temperature controller is provided to maintain the constant temperature. The ultrasound wave is also reflected and attenuated by foreign substrates or contaminated water; therefore, a debris filter and sterilizing devices are installed with the temperature controller in the instrument. Third, an automatic database program developed using LabVIEW enables the users to control instruments such as the function generator, oscilloscope, and temperature controller in order to reduce human error and load. The program also stores the result data automatically in the database to analyze the acoustic lens properties of the medical ultrasound transducers.

### 2.1. Fixture Stage

**Figure 3:**
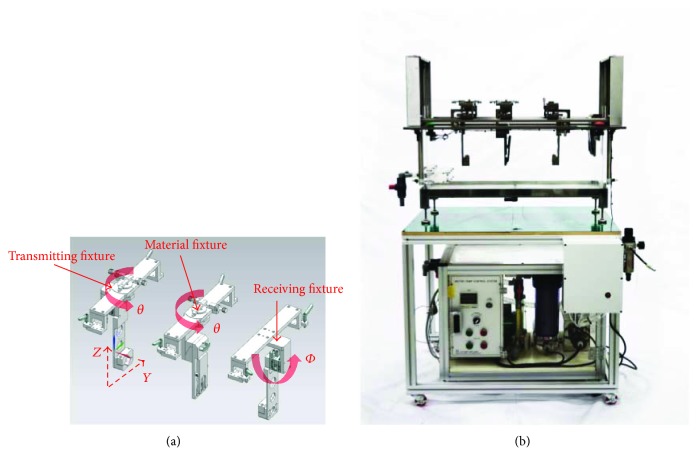
(a) Schematic diagram of the fixture stages (transmitting, receiving, and material fixtures) and (b) the fixture stages in the water tank.

As shown in Figure 3, the fixture stage is composed of the transmitting, receiving, and material fixtures. Firstly, the receiving fixture only fixes the receiving ultrasound transducer and can move the receiving transducer in the *Φ* direction. The transmitting fixture can move the transmitting transducer in the Z, Y, and *θ* directions. The material fixture must be placed parallel in the Y direction with the receiving fixture, only when the material fixture holds the testing material. After the material is placed in the material fixture parallel to the receiving fixture, the transmitting fixture will begin to align the material with the testing material. If only one transmitting fixture is used for alignment, it is more convenient to align the material with the instrument. Therefore, the transmitting fixture, which holds the transmitting transducer, can only be aligned in the *θ* direction with the material fixture and receiving fixture. This structure can compensate for the nonalignment situation of the testing material, and the transducers with the transmitting fixture can improve the signal-to-noise ratio of the receiving ultrasound waves. Our fixtures are adjustable, to minimize the unwanted reflection due to possible improper positioning from the fixture positions so that we can obtain the ideal characteristics of the testing materials such as very small size acoustic lens.

The velocity of the ultrasound wave can be generally changed by water temperature variation. Therefore, as shown in Figure 3(b), we used a temperature controller (MZ4M, Autonics, Busan, South Korea) to maintain the water temperature at 36.5°C because medical ultrasound transducers are typically utilized to monitor the human body. The pump (PM-150PM, Wilo, Dortmund, Germany) in the temperature controller circulates the heated water in the water tank.

### 2.2. Automation Program

**Figure 4:**
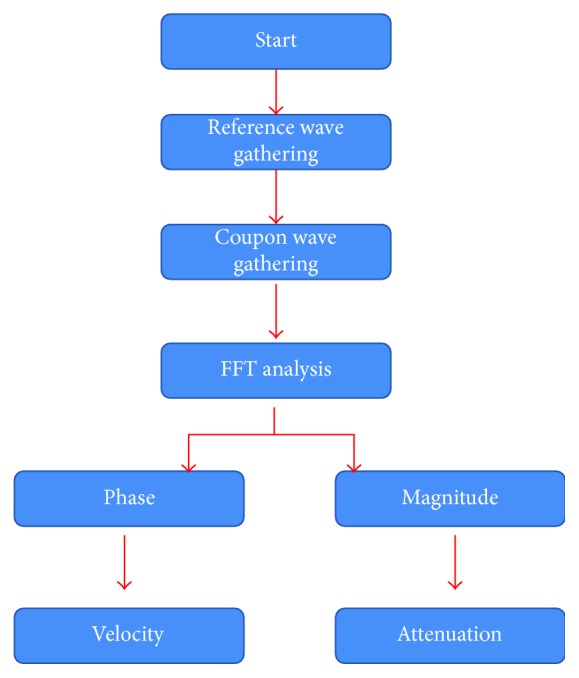
Automation program flowchart.

The measurement flowchart of the automation program is described in Figure 4. In the flowchart, the reference wave without the testing material is gathered, and then the coupon wave is collected in the oscilloscope when the testing material is placed in the material fixture. Using fast Fourier transform (FFT) analysis on the computer, the magnitude and phase of the coupon and reference wave are extracted to calculate the velocity and attenuation coefficient of the acoustic lens material in the medical ultrasound transducer. The developed program controls the function generator (AFG3101C, Textronics, Beaverton, OR) and oscilloscope (62xi-A, Teledyne Technology, Thousand Oaks, CA, USA) and shows the acquired data in the computer display. Additionally, the program stores and reviews the data using the mySQL database software tool. As previously described, this program gathers data using a GPIB-USB (NI GPIB-USB-HS, National Instrument, Austin, TX, USA), to analyze the velocity and attenuation coefficient, and maintains a constant water temperature with the temperature controller automatically. GPIB-USB-HS achieves IEEE 488.2 performances. The speed of IEEE 488.2 is 1.8 MB/s, which is enough to process the data in our developed instrument.

**Figure 5:**
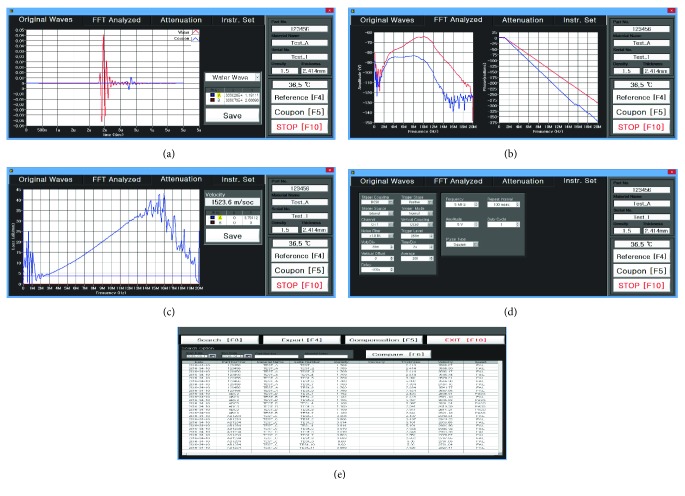
Automatic measurement program interface. (a) Original Wave panel, (b) FFT Analyzed panel, (c) Attenuation panel, (d) Instr. Set panel, and (e) ALDRP.

Figure 5 shows the graphical user interface of the program.  Figure 5(a) shows the “Original Waves” panel menu. The reference wave is shown when the ultrasound wave is generated from the transmitting transducer and then detected by the receiving transducer without passing through the testing material. Then, the coupon wave is shown when the ultrasound wave generated by the transmitting transducer passes through the testing material attached to the material fixture, and the attenuated ultrasound wave is then detected by the receiving transducer.  Figure 5(b) shows the “FFT Analyzed” panel menu. The spectrum data of the reference and coupon waves are processed using FFT. The magnitude and phase data of the reference and coupon waves are displayed, as shown in Figure 5(b).  Figure 5(c) shows the “Attenuation” panel menu. The calculated attenuation coefficient and velocity of the ultrasound waves (reference and coupon waves) are displayed, as shown in Figure 5(c).  Figure 5(d) shows the “Instr. Set” panel menu. This menu consists of the instrument settings that the user defines, such as trigger setup, delay, operating frequency, amplitude, pulse type, and duty cycle of the input signal. As shown in Figure 5(e), the acoustic lens data review program (ALDRP) is a tool that can search the date, part number, material name, serial number, density, and thickness of the testing material information and shows the results such as velocity in the database. The review program can provide the dataset as a report that users can easily use to search and compare different datasets. All the experimental setup data related to the reference and coupon waves can be stored in the database of the review program.

## 3. Measurement Results

### 3.1. Reference Wave Variations

**Figure 6:**
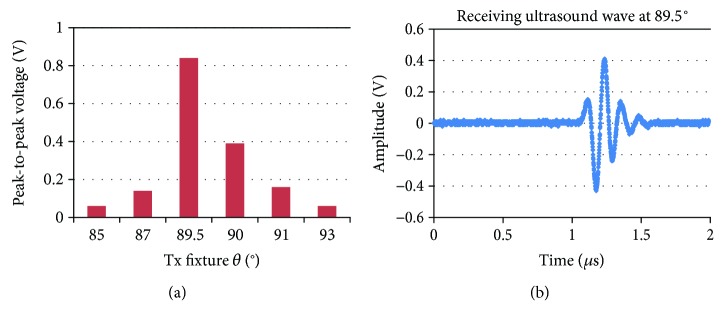
(a) Peak-to-peak voltage of the receiving ultrasound wave depending on the angle of the receiving fixture and (b) the aligned receiving reference wave at 89.5°.

In order to check the variations of the receiving reference wave according to the angle (*θ*) change of the transmitting fixture in the developed instrument, the reference wave was measured using two identical immersion transducers (V311-SU, Olympus, Shinjuku, Tokyo, Japan) installed in the transmitting and receiving fixtures. We also ensured that there was enough space to minimize the unwanted acoustic fluctuations due to multiple ultrasound interferences in the near field area, even though the distance between the transmitting and receiving fixtures could be changed with the programmed control [10, 22]. The angle change range of the transmitting fixture is between 87° and 93°. The resolution of the angle is 0.5°. The measurement result is shown in Figure 6(a).

According to the measurement result, the angle of the transmitting fixture is 89.5° when the highest peak-to-peak voltage is obtained. In Figure 6(b), the measured peak-to-peak voltage of the echo amplitude detected by the receiving transducer is 0.854 V when an 8 MHz and 10-V_p-p_ sine burst pulse was applied from the function generator. These results indicate that human or systemic torsion and errors can be mitigated by the fixtures in the instrument.

### 3.2. Instrument Repeatability

In order to check the instrument repeatability, the transducer (V311-SU) was used to measure the attenuation coefficient and velocity of a 2.54 mm epoxy acoustic lens material four times. Epoxy lens material was selected because the matching materials are one of the most typical acoustic lens material of the medical ultrasound transducers. The operating frequency ranges of the ultrasound wave were measured as 8 ± 2 MHz. The equation of the attenuation coefficient (*α*) is shown below [23].

(2) $$\begin{array}{c} \alpha =\frac{20}{t}\text{log}\left(\frac{A_{c}}{A_{r}}\right), \end{array}$$

where *t* is the thickness of the testing material [mm], *A*_c_ is the amplitude of the coupon wave, and *A*_r_ is the amplitude of the reference wave.

**Figure 7:**
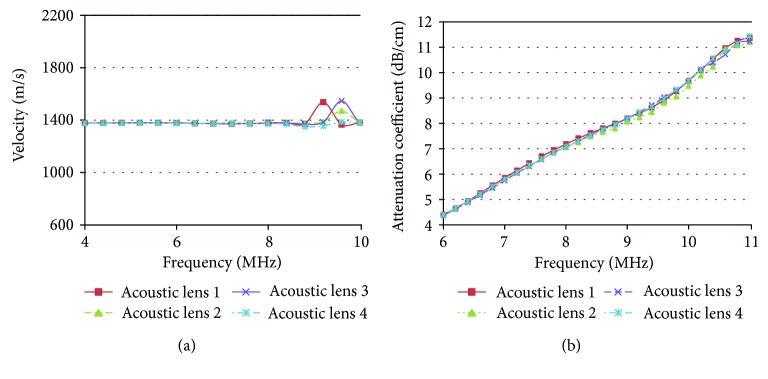
Measurement results of the (a) velocity and (b) attenuation coefficient. Acoustic lens 1, 2, 3, and 4 represent the calculated attenuation coefficient of the receiving ultrasound signal four times (acoustic lens 1, 2, 3, and 4 are represented by red solid, green dashed, purple solid, and blue dashed-dot lines, resp.).

Figure 7(a) shows the calculated attenuation coefficient values of the receiving ultrasound waves. In Figure 7(a), the average attenuation coefficients at 6, 8, and 10 MHz are 4.39, 7.10, and 9.62 dB, respectively. From the data in Figure 7(a), the calculated standard deviations are 0.01, 0.05, and 0.09 dB/mm, respectively. The velocity of the receiving ultrasound waves (*v*) is calculated [24] as follows:

(3) $$\begin{array}{c} v=\frac{1}{\left(1/v_{w}\right)-\left(\Delta \varphi \left(w\right)/\left(w*t\right)\right)}, \end{array}$$

where *w* and *t* are the width and thickness of the acoustic lens [dB/cm], respectively, Δ*Φ*(*ω*) is the frequency-dependent phase difference between the reference wave and coupon wave, and water velocity (*v*_w_) is the ultrasound velocity in water (1523.6 m/s at 36.5°C which is the average temperature of the human body).

Figure 7(b) shows the average velocity of the acoustic epoxy lens material. The average velocities at 6, 8, and 10 MHz frequencies are 1377.75, 1376.25, and 1382.00 m/s, respectively, and the standard deviations are 0.95, 2.62, and 1.41 m/s, respectively. These results also indicate that the maximum standard deviation of the attenuation coefficient and velocities of the receiving ultrasound waves at 6, 8, and 10 MHz frequency ranges are 0.21%, 1.10%, and 0.55% and 0.09%, 0.27%, and 0.14%, respectively. Therefore, the developed program provides accurate repeatability of the instrument.

## 4. Conclusion

Low-cost estimation instrument of the acoustic lens properties is developed herein to obtain precise acoustic lens data of medical ultrasound transducers. The simultaneous alignment between the fixtures can generate accurate acoustic lens data of medical ultrasound transducers with the help of the developed automatic database program for the instrument. The instrument improves the measurement repeatability of the ultrasound waves. The constant temperature, sterilization, and alignment between the transmitting and receiving fixtures and material fixture can be achieved, thus reducing human and measurement variation errors.

The fixtures were adjustable in any direction, and these fixtures could be supported by hardware such that the resolution of these fixtures was supported by the hardware. In particular, the transmitting fixture can control the angle (*θ*) to correct the nonalignment caused by a mechanical error of the fixtures or internal torsion of the constructed instrument because the excellent standard deviation values of the attenuation coefficient and velocity were confirmed as 1.10% and 0.27% at 8 MHz, respectively. A user interface program provides the function to be communicated between the function generator and oscilloscope in the instrument.

The developed review program's connected customized database can search and save various material information and store the experimental results automatically. The instrument helps to reduce the user's load. The developed database-supported program can control the function generator and oscilloscope automatically. It automatically calculates the results obtained from the instrument, and it helps the user to search and review the resulting data. The developed instrument described in this paper has recently been provided to Siemens Healthineers, Korea and USA, and it is currently used for their medical ultrasound research.
